# How Does Diet Influence Our Lives? Evaluating the Relationship between Isotopic Signatures and Mortality Patterns in Italian Roman Imperial and Medieval Periods

**DOI:** 10.3390/molecules26133895

**Published:** 2021-06-25

**Authors:** Marica Baldoni, Alessandra Nardi, Flavio De Angelis, Olga Rickards, Cristina Martínez-Labarga

**Affiliations:** 1 Centre of Molecular Anthropology for Ancient DNA Studies, Department of Biology, University of Rome Tor Vergata, 00133 Rome, Italy; marica.baldoni@gmail.com (M.B.); flavio.de.angelis@uniroma2.it (F.D.A.); rickards@uniroma2.it (O.R.); 2 Ph.D. Program in Evolutionary Biology and Ecology, Department of Biology, University of Rome Tor Vergata, 00133 Rome, Italy; 3 Department of Mathematics, University of Rome Tor Vergata, 00133 Rome, Italy; alenardi@axp.mat.uniroma2.it

**Keywords:** stable isotopes, hazard models, age at death, survivorship, molecular archeoanthropology

## Abstract

The present research investigates the relationship between dietary habits and mortality patterns in the Roman Imperial and Medieval periods. The reconstructions of population dynamics and subsistence strategies provide a fascinating source of information for understanding our history. This is particularly true given that the changes in social, economic, political, and religious aspects related to the transition from the Roman period to the Middle Ages have been widely discussed. We analyzed the isotopic and mortality patterns of 616 individuals from 18 archeological sites (the Medieval Latium sites of Colonna, Santa Severa, Allumiere, Cencelle, and 14 Medieval and Imperial funerary contexts from Rome) to compile a survivorship analysis. A semi-parametric approach was applied, suggesting variations in mortality patterns between sexes in the Roman period. Nitrogen isotopic signatures influenced mortality in both periods, showing a quadratic and a linear effect for Roman Imperial and Medieval populations, respectively. No influence of carbon isotopic signatures has been detected for Roman Imperial populations. Conversely, increased mortality risk for rising carbon isotopic values was observed in Medieval samples.

## 1. Introduction

Carbon and nitrogen stable isotope analysis from bone proteins is routinely employed in bioarcheological research to investigate dietary habits of human populations from different prehistoric and historical periods [1,2,3]. Such studies recently focused on investigating the relationship between diet, health status, and demographic parameters [4,5,6,7,8]. Undoubtedly, diet has played a pivotal role in our history [9,10,11], and it continues to do so in the life of all of us, while also representing an important factor influencing health [12]. In modern society, we are warned not only about the necessity of pursuing global sustainable dietary programs, considerable store is given to the fact that following a healthy diet may help in preventing chronic pathologies [12,13]. The relationship between diet and death risk, analyzed in the research by Zazpe and colleagues [12] in a cohort of Spanish adult individuals, suggested differing mortality risks associated with specific dietary habits. In particular, individuals following a Mediterranean diet showed a reduced mortality risk [12]. Similar results were also observed in aged Italians [12,14].

The isotopic signatures of bone proteins, reflecting the isotopic composition of protein nutritional sources of the last years of an individual’s life, have been extensively reported in the literature [2,5,6,15,16,17,18]. With respect to dietary reconstructions, the carbon isotopic ratio (δ^13^C) makes it possible to discriminate between terrestrial and marine protein sources. Within the terrestrial ecosystem, it can distinguish C_3_ from C_4_ plant sources; on the other hand, the nitrogen isotopic signature (δ^15^N) is useful for determining the trophic level of the analyzed individual(s) [1,2,18,19]. It would be worth analyzing coeval plant and animal specimens along with human samples in order to better interpret the ecological context and to evaluate possible variations of the isotopic values due to specific agriculture and breeding practices, and/or to the analyzed sites’ geographical location and ecology [20,21,22,23,24,25,26,27,28,29,30].

Despite being a valuable proxy for investigating past human diets, bulk stable isotope analysis can only discern macro food groups. As such, it fails to reflect different nutritional routing of the micronutrients, and consequently it is incapable of directly linking changes in diet to health issues [31]. However, the multifaceted aspect of isotopic diet reconstruction requires further consideration; multiple lines of evidence should be incorporated so as to provide a reliable marker for discerning the health of past populations. Indeed, isotopic values may also vary in response to both a high protein diet and as a consequence of metabolic/nutritional stress and pathological conditions of the individual [6,7,8,31,32,33,34,35,36,37,38].

**Figure 1:**
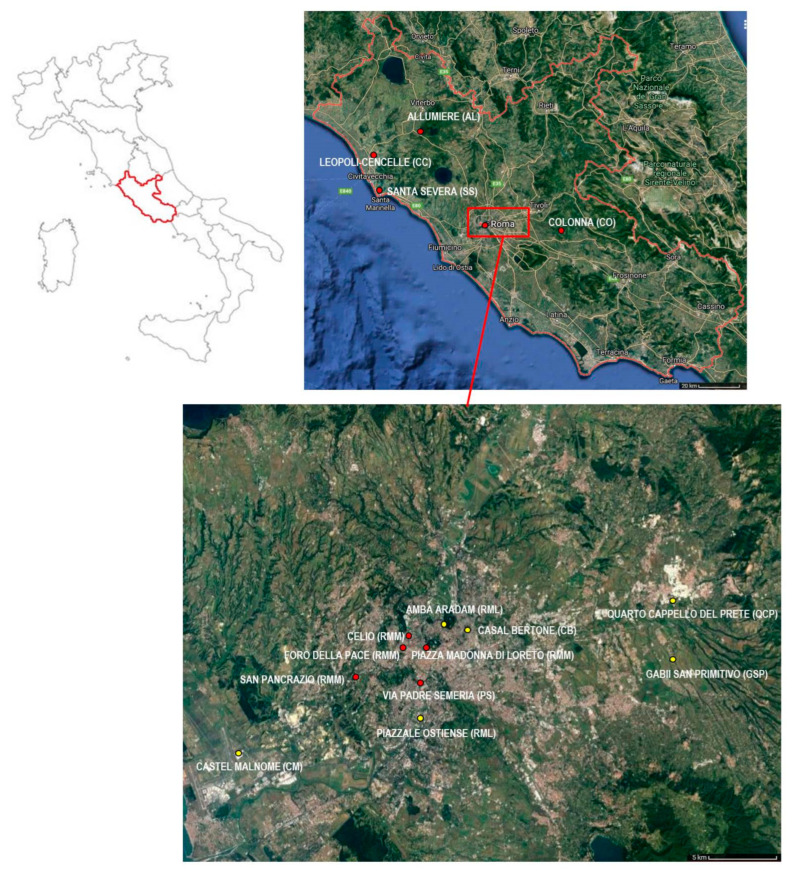
Location of the Latium region and of the Roman Imperial (yellow dots), and Medieval (red dots) sites analyzed in the present research (modified from https://www.google.it/maps, accessed on 19 March 2021).

Recently, Redfern and colleagues [5] offered new perceptions on isotopic analysis regarding demographic data in order to evaluate the relationship between diet and mortality in Roman Britain. The new approach proposed by the authors [5] made it possible, in our opinion, to extend the use of stable isotopes to paleodietary reconstructions, with their research applying stable isotopic data for investigating complex population dynamics. Accordingly, we aimed to investigate the relationship between diet and survivorship in a Mediterranean ecological setting, focusing on the Roman Imperial and Medieval periods. To better understand the relationship between diet and life-span in the Roman Imperial and Medieval periods, we considered 616 individuals from 18 Roman and Medieval archeological sites from Latium (Figure 1), which had previously been analyzed at the Centre of Molecular Anthropology for ancient DNA Studies at the Department of Biology of the University of Rome “Tor Vergata” [39,40,41,42,43,44,45]. As explained in detail in the Materials and Methods’ section, however, our research followed a semiparametric statistical model which did not assume a specific shape for the mortality curves.

### 1.1. Dietary Pattern in Imperial Rome

Roman diet reconstruction is currently an interesting research field, even though the historical sources have provided plentiful evidence of the wide range of foods of the Roman people. Latin literature and art both feature the diet of ancient Romans [46,47], dating from the Republican period. Among the earliest sources dealing with the topic, Cato’s *De Agricultura* and Marcus Terentius Varro’s *De Re Rustica* are the most renowned, while Columella is considered one of the first authors writing about agricultural practices and food processing for the Imperial period. His *De Re Rustica* is still considered a prime source of information for framing the diet in that period. Pliny the Elder’s *Naturalis Historia*, meanwhile, puts the Imperial diet in a historical perspective by describing in detail faunal and horticultural Roman landscapes.

A thorough depiction of the Roman cooking styles is found in Petronius’ *Satyricon* and Apicius’ *De Re Coquinaria*, with the latter collecting hundreds of recipes showing the foodstuffs available to the wealthy Romans. As well as literary sources, iconographical representation of food was also widespread in the Roman time as a decoration motif for rooms associated with food consumption in wealthy estates, whether in the form of floor mosaics or wall paintings [48].

According to this evidence, grain would have constituted the primary food for Romans. Carbohydrates from grains would in fact have represented about 70% of their daily energy intake [49]. Grain was used mainly as puls, a kind of soup also combined with vegetables, meat, and cheese [50]. Cereals were one of the Empire’s most important crops, and grain was imported in substantial quantities from Sicily and Egypt. Accordingly, the Edict of Diocletian regulated the grain trade and also defined the maximum price of wheat, barley, and millet. The pivotal role of cereals in the Empire led to the development and refinement of agricultural and artificial farming practices, as well as food preservation techniques [51] to maintain a continuous supply of these foodstuffs. However, among cereals, the use of millet is still questionable: It might have been mainly used as fodder for stocks rather than for human consumption [52], even if its use for medication purposes was mentioned by classical authors such as Dioscorides.

Despite the widely documented consumption of cereals, the Roman diet was also based on the consumption of vegetables, fruits and pulses [50,53].

Meat, for its part, was a vital element in diet. Livestock breeding and trade flourished [54,55], with goats, sheep, lambs, and pigs being dietary favorites [55,56].

Fish consumption, however, is less well-defined; fish was alternatively seen as either a luxury or as an everyday staple [46], depending on the local contexts. In a oversimplified perspective, preserved and fresh fish was consumed mostly by the elite [57], even though *garum,* famously a staple of the legionnaire food kit, widens this evaluation.

Reliable insights into the Roman diet can also be obtained from increasing archeobotanical evidence such as that documented by previous research at Pompeii and Herculaneum [58], or by the evaluation of the food remains and their constituent compounds trapped in the calcified matrix of dental calculus [59,60]. Similarly, recovered faunal remains suggest which kinds of meat and fish were available [61,62,63,64].

An equally critical contribution towards understanding the diet of Ancient Romans is represented by the analysis of human skeletal remains.

Indeed, despite the multiple sources about the ancient Roman diet, such direct physical evidence is fundamental for reconstructing foods consumed by ancient Romans. Several research examples have allowed for reconstructing this diet in western populations of the *Suburbium* by stable isotopic data [63,64,65,66] or those buried in Christian catacombs [67,68,69]. Moreover, further studies also provided important evidence about dietary reconstruction of common people living close to the city walls [16,17,70]. Recently, more data has been added [42,71], which allowed for a more in-depth analysis regarding the diet of commoners.

The overall dietary landscape turned out to be surprisingly heterogeneous and revealed the multifaceted aspects of the capital of one of the most powerful empires in Antiquity.

The available isotopic data confirm that C_3_ plants represented a stable food source for Romans, especially to the lower social classes. C_4_ plants also seem to have been consumed, although this practice seems less widespread and only a few isotopic examples of evidence have been found for it—perhaps due to local-exploitation [16]. Despite the use of administrative grain supplements to sustain a part of the population, the location of the settlements (and consequently of the necropolises) favored the preferential consumption of produce grown locally.

It is clear that supplying the Roman Imperial population with food was far from easy, above all considering its ever-increasing size [16,17,42]. Furthermore, it is worth considering that living in an urbanized and heterogeneous context such as “*Urbs*” and its *Suburbium* may have also had an impact on dietary preferences [42]. Moreover, it should be borne in mind that people were not allowed to be buried inside the city walls, therefore the Imperial Rome necropolises’ locations needed to be established in the *Suburbium* [16].

Farming, hunting, fishing, and/or breeding produced the vegetables, meat, and cheese to meet the feasible protidic intake needed [16,17,42,63,64,65,70]. Remarkably, fish exploitation increased in preference among early Christians [67]. Only a few people across all the studied samples could be said to consume steady and sizeable marine and/or freshwater resources.

In short, the intricacy of Roman society and its trade during the Imperial period accounted for the access to an increasing variety of foods [66]. A portrait of Roman dietary habits remains fascinatingly incomplete.

### 1.2. Dietary Patterns during the Middle Ages

Descriptions of Medieval diet, recipes, and medical indications may be retrieved in historical sources, as clearly outlined by Montanari [72], with these amply supported by material data and archeozoological findings. Again, isotopic evidence plays a pivotal role [39,40,41,43,44,45,73,74,75,76,77]. Overall, diet in the Medieval period was characterized by the mutual influence of the Mediterranean and Celtic/German food habits [78,79].

The two nutritional regimes were extremely different. The former was mainly centered on the pattern grain-oil-wine; on the other hand, the dietary patterns of Celtic and Germanic populations were less dependent on cereals, with diet being mainly based on hunting and fishing products as well as on wild fruits [79,80,81]. These foodstuffs were often accompanied by meat from breeding animals and vegetables from small, cultivated plots [79,80]. The transition from the Roman to Medieval period also led to changes in the exploitation of the environmental sources and spaces [79,82,83,84].

The fall of the Roman Empire and its centralized power led to a dramatic variation of the production systems, with these becoming increasingly localized. This shift, combined with the progressive expansion of Germanic populations, determined the variation of food-webs and dietary habits among Southern European populations [9,45,79,81,83]. Meanwhile, the Mediterranean dietary program was expanded towards Northern regions, too, mainly thanks to the ecclesiastic sponsorship of products like bread, wine, and oil [79]. Therefore, the Medieval diet resulted from the mutual influence between the two programs and their merging in a new model [78,79].

The Medieval diet was rich in animal products, both meat and fish [79]; pork meat became a dietary favorite, along with, in the Early Medieval period, game-meat derived from hunting [79,81]. Remarkably, an increasing preference for riverine resources (e.g., freshwater fish) rather than marine products characterized the Middle Ages [79]. Sorghum, millet, oat, and spelt were the staple grains, particularly for lower-middle classes [40,43,75,79,85,86,87]. Indeed, differences in dietary habits characterized class divisions as a whole, as did environment and culture [72,77,79,88]. Accordingly, Central and Southern Italy maintained a more Mediterranean dietary plan characterized by the residual consumption of wheat, barley, ovine, and marine resources, due to those sites’ locations and supply availability [77,79,89,90].

It should also be considered that the Medieval period comprises several centuries when the dietary trajectories and evolution were subject to adjustments. In particular, the transition from Early to Late Middle Ages saw a sharp dietary variation as the availability of animal-derived products dramatically reduced for the non-elite classes, who were forced to develop a dietetic plan mainly based on cereal consumption [91,92]. As a consequence, grain-derived products such as bread, mainly produced from C_4_ cereals, rapidly became widespread for lower class people, with cereals providing over half of their nutritional intake [92,93].

The less expensive cereal-based diet also spread in Northern regions between the 11th and 13th centuries to cope with population growth and consequently increasing demand [90]. A reduction in meat consumption was also typical for ecclesiastic movements [81,94], as Catholic practices compelled abstinence from meat according to the calendar, with fish consumption being provided as a substitute [43,75,81,87,94,95,96,97,98,99,100,101,102,103,104,105].

On the basis of the background of the analyzed skeletal collections, the aim of the present research was to directly analyze whether the extensively documented differences between the two periods could also be observed in a specific context such as the Latium Region. We expected to detect differences in dietary patterns between Imperial Roman and Medieval populations and posited that these variations would impact on individuals’ survivorship. Indeed, although the dietary habits of Roman Imperial and Medieval people have been widely described, it is the human skeletal remains (in this case, carbon and nitrogen stable isotope analysis from bone proteins) which provide the direct and conclusive evidence of their dietary patterns.

## 2. Results

The analyzed sample consisted of 616 individuals from 18 archeological sites in Latium (Italy) dating back to Imperial Roman and Medieval periods (N = 212, N = 404, respectively) for which we performed a comparative analysis between both periods, exploring the complex relationship among isotopic values, sex, and age at death.

In the Roman Imperial group, the majority of the samples (N = 199) date back to the 1st–3rd centuries CE. A few observations (N = 13) date back to the 4th–5th centuries. However, these were considered together with the Imperial Roman samples due to the smallness of the sample, the chronology (4th–5th centuries CE; [45]) and the similarity to the Roman funerary context, leaving other historical particularities aside [17,106].

**Figure 2:**
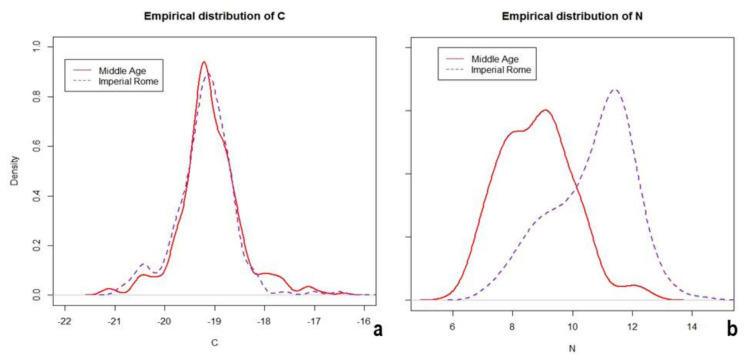
Empirical distribution of δ^13^C (**a**) and δ^15^N (**b**) in Roman and Medieval analyzed populations from Latium (Italy).

Observed levels of δ^15^N in Roman and Medieval periods were significantly different (*p* < 0.0001; Figure 2): median values were 11.0‰ (Q1 9.7‰; Q3 11.7‰) and 8.8‰ (Q1 7.8‰; Q3 9.6‰), for Roman Imperial and Medieval populations, respectively.

However, no significant difference exists for δ^13^C (*p* = 0.1405): median levels were −19.2‰ (Q1 −19.5‰; Q3 −18.9‰) in Romans and −19.1‰ (Q1 −19.4‰; Q3 −18.8‰) in Medieval populations. As the sex ratio did not differ substantially between the two groups (females’ percentages were 37.5% in Imperial Rome and 37% in the Middle Ages; *p* = 0.9901), we explored the isotope value distribution stratified by sex.

**Figure 3:**
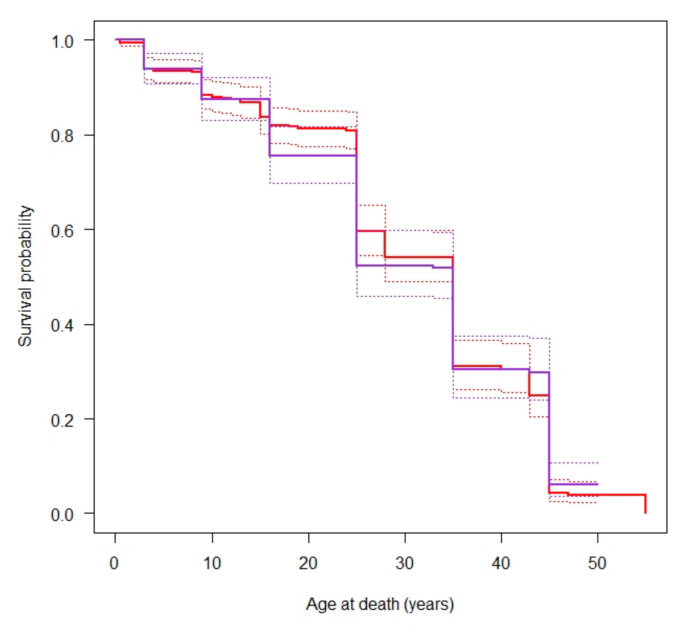
Survival curves for Imperial Roman (blue) and Medieval (red) populations with confidence bands (dashed lines).

Estimated survival curves were similar (*p* = 0.6679) in the two periods (Figure 3), and the median age at death was 35 years in both.

Multivariable analysis was based on the Cox model: a detailed description of the characteristics of this model is presented in the Materials and Methods’ section. Table 1 shows risk factors affecting age at death at Cox multivariable analysis: parameter estimates and corresponding hazard ratios (HR) with confidence intervals are reported.

**Figure 4:**
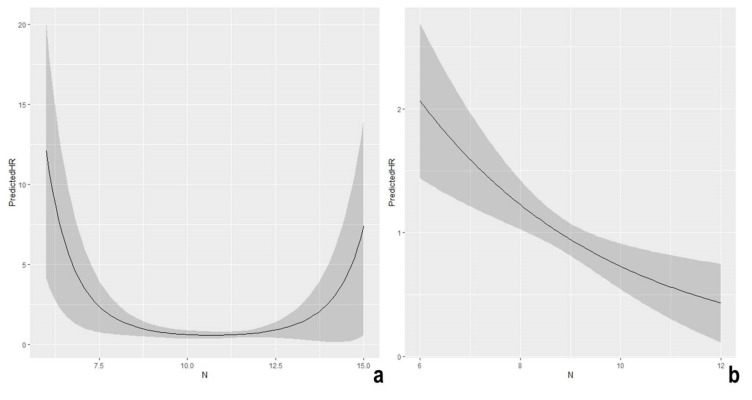
Shape of the predicted Hazard Ratio (HR) as a function of nitrogen isotopic signatures (δ^15^N) in the Imperial Rome (**a**) and in the Middle Ages (**b**). Sex was set to “males” and carbon values to the median value.

Mortality varied according to changes in nitrogen isotopic values in both the Roman and Medieval periods. In particular, Roman Imperial populations are characterized by a non-linear effect with an increased predicted Hazard Ratio (HR) for δ^15^N lower than 8‰ and greater than 13‰ (Figure 4a).

Conversely, increasing levels of δ^15^N were associated with a decreasing risk of death in the Middle Ages (Table 1); a reduction of 23% was estimated for the unitary increase of nitrogen signatures (HR = 0.77, Table 1) (Figure 4b). Here, no departure from linearity was observed, although it is worth mentioning that δ^15^N levels in the Medieval period were lower than 13‰.

**Figure 5:**
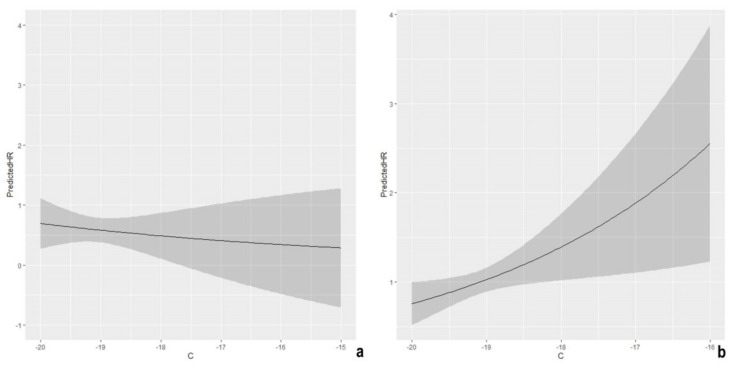
Shape of the predicted Hazard Ratio (HR) as a function of carbon isotopic signatures (δ^13^C) in the Imperial Rome (**a**) and in the Middle Ages (**b**).

No significant effect was detected for δ^13^C in Roman Imperial populations (Figure 5a). At the same time, a different scenario was depicted for the Medieval period where increased mortality was observed with rising levels of δ^13^C with an estimated HR of 1.36 (Figure 5b).

Empirical distributions of δ^15^N and δ^13^C were similar for males and females in both periods (Supplementary Figure S1). In detail, in the Imperial Roman period, median isotopic values were −19.1‰ and −19.3‰ for δ^13^C and 11.0‰ and 10.9‰ for δ^15^N in males and females, respectively. Isotopic median values in the Medieval period were −19.1‰ and −19.1‰ for δ^13^C and 8.8‰ and 8.9‰ for δ^15^N in males and females, respectively.

**Figure 6:**
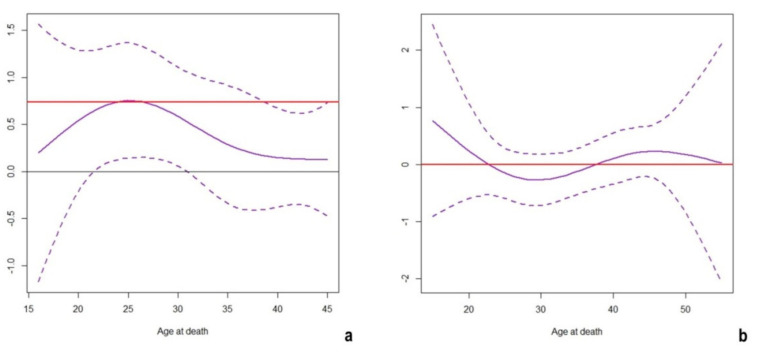
Estimated Hazard Ratio (HR) logarithm of females vs. males over time (95% confidence band) in Roman Imperial (**a**) and Medieval (**b**) analyzed populations. The red line corresponds to the estimated parameter (reported in Table 1), assuming it as constant.

A significantly increased mortality characterized the Roman females with respect to the males (*p* = 0.0032), with an estimated HR of 2.09. Notably, this effect was not constant throughout the female individuals’ lifespan, showing an increased risk for females at about 25 years (Figure 6a). By contrast, no significant variations in mortality risk were observed between sexes in the analyzed Medieval populations: note that the confidence band always includes 0 (Figure 6b).

## 3. Discussion

These results seem to confirm the well-known heterogeneity between the Roman Imperial period and the Middle Ages. Despite this heterogeneity, a relationship between nitrogen isotopic signatures and mortality risk were observed in both, although with different magnitude and trends.

The quadratic effect observed in Roman Imperial populations and expressed as the “U” curve in Figure 4a, is in line with the current knowledge of nitrogen isotopic signatures and their usefulness in bioarcheological research. Even though it is well established that different dietary habits could result in heterogeneous isotopic reconstructions, the physiological aspects seem to contribute to the individual signature.

In healthy individuals, δ^15^N values indicate their dietary protein sources. However, pathological/stress conditions decouple the balance between nitrogen excretion and protein synthesis [7,8,34,36,37]. In these situations, the individual is characterized by a negative nitrogen balance, meaning that ^14^N is preferentially excreted, thus resulting in increased ^15^N enrichment of body tissues’ proteins [31,36,107]. Basically, δ^15^N in body tissues varies according to the individual’s position in the trophic chain, but a high nitrogen isotopic ratio may also be a clue for the disruption of individual health [1,2,6,7,8,31,32,33,34,35,36,37,43].

To date, it is not surprising that mortality risk in Roman Imperial populations increases at the two ends corresponding to the lowest (δ^15^N < 8‰) and highest (δ^15^N > 13‰) nitrogen values. The increase in mortality for individuals returning low nitrogen values may be due to an inadequate dietary intake or a diet based on low protein sources. Many studies, in fact, emphasize that suboptimal nutrition may eventually alter the correct functioning of the individual’s immune system [6,108,109,110,111]. Adequate diet and suitable levels of micronutrients are important for controlling inflammations and oxidative stress as well as for maintaining the levels of antibody production and, more in general, for the correct functioning of the immune response [109,112].

Feeding the Roman Imperial population was challenging; Rome was then one of the world’s most crowded cities [16,17,42,113,114] and its heterogeneity [113] was magnified by social stratification [16,17,42,115].

Accordingly, lower social classes probably would not have had an adequate dietary intake. Furthermore, despite the well-known triad (grain-oil-wine) on which Romans based their diet, there is no consensus as to the extent of their meat and fish consumption [16,17,42,63]. The analyzed populations were—at least with regards to the locations of the burial sites—in the suburban areas [42]. Life conditions in such an urbanized context as the city of Rome may have impacted significantly on mortality [50,114]. Furthermore, the analyzed individuals’ dietary intake was probably not sufficient for the biomechanical load they were subjected to, thus reducing their life expectancy [42,116].

Previous research on non-specific stress markers [117] suggests that multiple markers such as *cribra cranii*, *cribra orbitalia*, tooth enamel hypoplasia, periostitis, and—although less diffused—Harris lines were widespread in the Roman samples. The analyzed skeletal series were also extensively affected by several oral health-impairment markers such as caries, alveolar retraction, *ante-mortem* tooth loss, and dental calculus [117]. Overall, the bioarcheological analysis [116,117] confirms that the individuals probably belonged to low social strata, representing a group subjected to the interplay of harsh living conditions and inadequate diet.

The lower nitrogen values observed in Medieval populations with respect to the Roman period suggests a reduction in animal products’ consumption (8.8‰ vs. 11.0‰; *p* < 0.0001). This evidence appears to conflict with respect to the historical description of Medieval food habits [79,80,81]. However, local variations were observed in Central and Southern Italy [77,79,89,90]. It is well-known that the fall of the Roman Empire also resulted in the collapse of the central economic power behind the development of autonomous local societies and economies [45,118]. Furthermore, it is conceivable that the collapse of the centralized economy would have impacted more on urban contexts than on peri-urban and/or rural communities experiencing different lifestyles and dietary habits, even during the Roman Empire [16,17,42,63,80].

In the analyzed Medieval populations, the isotopic evidence suggests diet was predominantly based on terrestrial food-webs, including both plant and animal protein sources. However, with a median δ^15^N of 8.8‰ (Q1 7.8‰; Q3 9.6‰) it is reasonable to suppose that the animal-derived products (both meat and dairy) did not have a predominant role in these individuals’ subsistence. We mainly refer to terrestrial animals, as fish consumption probably did not play a pivotal role in the subsistence of these populations [39,40,41,44,45].

The absence of δ^15^N values higher than 12.7‰ in Medieval samples could be due to the analyzed individuals enjoying more healthy conditions and/or to a bias in sampling. Whichever is the cause of the absence of extremely high nitrogen ratios, it is not possible to make inferences on the impact of high nitrogen values on mortality in Medieval populations.

The absence of relationship between the carbon isotopic signature and the mortality pattern in Roman Imperial populations is not particularly striking. Grain represented one of the cornerstones of the Imperial diet [16,17,42,63,82,84]. Although evidence of C_4_ plant intake has been suggested by some researchers [16,17,63] these cereals were less diffused in the dietary plan of Imperial Romans [16,17,42,63]. Isotopic data of the analyzed populations suggest that C_4_ plant consumption could be hypothesized for some of the individuals [42].

A different scenario was depicted for the Medieval period in which mortality risk increases for higher (less negative) δ^13^C values. C_4_ plants started becoming a more popular foodstuff at the Medieval table, especially for people belonging to lower social strata [75,79,84,87]. Although this trend seems not to hold for the Latium region, some evidence of C_4_ plant consumption has been observed in the analyzed populations. Isotopic ratios (δ^13^C), ranging from −21.2‰ to −16.4‰, and the presence of extreme carbon values (δ^13^C > −18.0‰), however, suggest that C_4_ plants were probably not consumed by the whole population (at least not in quantities that allow shifting the isotopic signal). This may explain the width of the confidence bands observed in Figure 5. Isotopic values were also supported by archeobotanical analyses on dental calculus suggesting the consumption of C_4_ plants in some individuals from Santa Severa [43], Colonna [85], and Allumiere [40]. These findings, along with a moderate shift in isotopic signatures, suggest that the analyzed individuals effectively consumed these plants, although to what extent exactly the retrieval of starches and/or macroresidues in dental calculus occurred is not clear [43,85]. Plants grounded on the C_4_ photosynthetic pathway generally have lower nutritional values than C_3_ plants [119], even though in recent years, increasing attention has been paid to the impact of current climatic changes in the mineral and protein content of C_3_ crops [119,120,121,122,123,124,125,126]. In a Middle Ages context, however, the consumption of foods with lower nutritional values may have lowered life expectancy. Another aspect to be borne in mind is that severe and/or systemic health issues such as infections or metabolic disruptions could trigger a bodily response, which may alter the carbon isotope fractionation, with a consequent rise of the δ^13^C of forming tissues [127]. Thus, the analysis of bulk collagen data in frail people could result in higher δ^13^C values, which could thus account for the increased mortality.

Based on the obtained results, diet therefore seems to have a significance in survivorship of both Roman and Medieval populations from Latium (Italy).

Another factor with a role in mortality patterns related to isotope signatures is sex. While, apparently, no differences in mortality risk have been observed in Medieval populations (Figure 6b), this could be a bias in sampling strategies. In fact, the percentage of females who died between 20 and 30 years in Imperial populations is higher with respect to males (females aged between 20 and 30 years: 24/56 = 42.86%; males aged between 20 and 30 years: 21/88 = 23.86%). Instead, an almost equal percentage has been observed for males and females that died at between 20 and 30 years of age in the Medieval populations (females aged between 20 and 30 years: 27/89 = 30.34%; males aged between 20 and 30 years: 53/150 = 35.33%). As the sexes are almost equally represented, it is currently impossible to make any inference on the effect of sex on mortality in the analyzed Medieval populations.

The observed higher risk for females in the Imperial period could more specifically pertain to individuals of childbearing age. It is known that pregnancy and childbirth could threaten women’s health and even impact their survivorship [128,129,130]. The impact was probably not only derived from problems occurring during pregnancy; it may also be a consequence of multiple factors causing progressive weakness in young female individuals to *pre-* or *post-partum* in general [128,129]. The research by De Angelis and colleagues [71] reports isotopic evidence concerning newborns breastfed until the age of three. However, infant growth also requires an adequate nutritional intake for the lactating mother or wetnurse [131,132,133] that may have been lacking in past populations. Although no differences in isotopic signatures have been observed between sexes, the exact interpretation of this result is limited; indeed, based on the isotopic data, males and females in the analyzed samples followed similar dietary habits, even though it is not possible to ascertain if they also received the same servings and/or consumed the same cuts of meat [101]. While one may hypothesize many factors impacting young females’ mortality, it is actually impossible to disentangle the real influence of one factor from another.

## 4. Materials and Methods

Following the approach proposed by Redfern and colleagues [5] (2019), a semi-parametric approach was applied for a comparative analysis of Roman (N = 212) and Medieval (N = 404) populations aiming at evaluating the effect of diet (in terms of C and N isotopic signatures) and sex on mortality in the two periods.

The data were collected for published surveys from the Centre of Molecular Anthropology for ancient DNA Studies of the Department of Biology of the University of Rome “Tor Vergata” for which individual carbon and nitrogen isotope values, sex, and age at death data were available (Table 2). We selected only our published data in order to avoid any bias related to differences in the applied protocols for isotopic analysis from bone proteins.

We are aware that the individuals from Amba Aradam (AA) and Piazzale Ostiense (PO) date from the transitional period prior to the fall of the Roman Empire. However, the archeological characteristics of the funerary contexts led us to include them in the Imperial cluster.

As stated, for the analyzed specimens, data on sex and age at death assessment were available. Age at death estimation was based on different criteria for adult and non-adult individuals, respectively. In particular, for adult individuals, changes in the auricular surface of the ilium [134], on the pubic symphysis [135], and on the sternal end of the fourth rib [136,137] were observed. The degree of obliteration of the cranial sutures [138] and of dental wear [139,140] were also considered.

For infants and adolescents, the estimation of age at death was instead based on the degree of tooth formation and eruption [141], on long bone diaphysis and clavicle length measurements [142,143,144], as well as on the observation of the development of primary and secondary ossification centers [145].

Sex assessment was performed only on adult individuals. In this case, the morphological analysis followed the criteria proposed by Acsàdi and Nemeskeri [146], by Ferembach and colleagues [147] and by Phenice [148].

### Statistical Analysis

In the first, descriptive step of the statistical analysis, continuous variables such as isotope values were described by median, first, and third quartiles, since some showed a skewed distribution with significant departure from the normal density. Kernel density estimates were utilized to describe empirical distributions.

Categorical variables were described by absolute frequencies and percentages. We used the χ^2^ test for categorical variables and Wilcoxon rank-sum test for continuous variables to compare groups.

Age at death was analyzed in the framework of survival analysis. In the case a reliable age at death estimation was not possible, observations were considered to be censored.

Unadjusted survival curves were estimated using the Kaplan–Meier method. The log-rank test was used to compare groups.

During multivariable analysis, the influence of δ^13^C, δ^15^N, and sex on the age at death was assessed through a semi-parametric approach chosen based on Cox’s model [149]. Let z be the vector of explanatory variables which includes in our study values of δ^15^N, δ^13^C and sex.

Cox’s model assumes that the hazard of death at age t can be expressed as

(1) $$h\left(t;z\right)=h_{0}\left(t\right)e^{\beta^{t}z}$$

or, equivalently, as

(2) $$\log\;\frac{h\left(t;z\right)}{h_{0}\left(t\right)}=\beta^{t}z.$$

In these expressions, $h_{0}\left(t\right)\;$ is the baseline hazard function for a reference subject with *z* = 0. We arbitrarily assumed as our reference individual a male with values of δ^13^C and δ^15^N set to their means in the corresponding period.

In this model, interest focuses on the vector *β* of regression parameters describing the effect of the explanatory variables on the log hazard ratio. For a single parameter *β_j_*, $e^{\beta_{j}}$ is the hazard ratio for a unit increase in *z_j_*, while $(e^{\beta_{j}}-1)\;$ can be interpreted as the percentage change in hazard. This is the parametric component of the Cox’s model. Regression parameters were estimated by maximizing the partial likelihood function [150] without making any assumptions about the shape of $h_{0}\left(t\right).$ This baseline hazard was regarded as a nuisance, infinite dimensional, parameter that constitutes the non-parametric part of the model. This feature characterizes Cox’s model and guarantees that estimates of covariate effects are not influenced by the chosen parametric model.

Note, however, that this model relies on the assumption of proportional hazards, e.g., the effect of covariates was assumed to be constant over time.

Several tied ages at death were observed and were handled using the exact partial likelihood method in our analysis.

In the Cox’s model, the effect of continuous variables was explored as such and initially modeled as linear. This assumption was verified by plots of deviance residuals [151] against covariate values. Where departures from linearity were detected, different parametric transformations were considered, including first and second degree fractional polynomials. A quadratic effect was detected for δ^15^N in the Roman period and added in the predictor of Cox’s model. Possible violations of proportionality were evaluated by plots and test statistics based on Schoenfeld residuals [152,153]. Time-dependent effects were modeled using penalized spline functions [154]. Note that violation of proportionality was detected for the effect of sex in the Roman period.

All analyses were undertaken using SAS version 9.4 (SAS Institute, Cary, NC, USA) and R version 4.0 (R Core Team (2012).

## 5. Conclusions

The present research represents the first attempt, to our knowledge, to investigate the relationship between carbon and nitrogen stable isotopic signatures and mortality patterns in Roman Imperial and Medieval populations from Latium (Italy).

Our results are consistent with the idea that diet influenced both Imperial and Medieval populations, although with different apparent trends. Imperial Roman populations’ mortality was significantly affected by extreme nitrogen values (both lowest and highest) and sex, with females at childbirth age experiencing a higher mortality risk than males. These results seem to reflect the complex, challenging, and heterogeneous living conditions that characterized Imperial Rome and its *Suburbium*.

Conversely, in Medieval populations, survivorship was impacted by decreasing nitrogen values and increasing carbon isotopic signatures. The current research not only aimed to disentangle the effect of several variables on mortality patterns, but also confirmed the importance of stable isotope analysis in bioarcheological research. This technique is already employed for reconstructing past populations’ dietary habits; here, it also could be extended to offer new insights into populations’ dynamics.

This approach could pave the way to further research involving not only Latium, but the whole of Italy and other European populations.

## Figures and Tables

**Table 1:** Results from the Cox’s model. The significance threshold was set to *p* = 0.05. The reference level for sex was “Male”. In the case of a linear effect, the Hazard Ratio (HR) was intended for increasing units.

Roman Imperial Period					
Variable	Parameter Estimate	Hazard Ratio (HR)	95% Hazard Ratio Confidence Limits		*p*
			Lower	Upper	
Sex	0.74	2.09	1.28	3.42	0.0032
(Female vs. Male)					
δ^13^C (x increasing unit)	−0.18	0.84	0.53	1.32	0.4440
Linear effect					
δ^15^N (x unit)	−2.93				0.0199
Linear effect					
δ^15^N (x unit)	0.14				0.0235
Quadratic effect					
**Medieval Period**					
**Variable**	**Parameter Estimate**	**Hazard Ratio** **(HR)**	**95% Hazard Ratio Confidence Limits**		***p***
			**Lower**	**Upper**	
Sex	−0.26	1.00	0.7	1.43	0.9875
(Female vs. Male)					
δ^13^C (x increasing unit)	0.30	1.36	1.04	1.77	0.0248
Linear effect					
δ^15^N (x increasing unit)	−0.26	0.77	0.67	0.89	0.0005
Linear effect					

**Table 2:** Summary of the analyzed samples dating to Roman Imperial and Medieval periods. For each site chronology, abbreviation and references are reported.

**Roman Imperial Samples (1st–5th Centuries CE)**			
**Archeological Sites**	**Chronology (Centuries)**	**Abbreviations and Grouping**	**References**
Castel Malnome	1st–3rd centuries CE	CM	[42]
Via Padre Semeria	1st–3rd centuries CE	PS	[42]
Quarto Cappello del Prete	1st–3rd centuries CE	QCP	[42]
Casal Bertone Necropolis	1st–3rd centuries CE	CBN	[42]
Casal Bertone Mausoleum	1st–3rd centuries CE	CBM	[42]
Casal Bertone Area Q	1st–3rd centuries CE	CBQ	[42]
Piazzale Ostiense	4th–5th centuries CE	RML	[45]
Amba Aradam	5th century CE	RML	[45]
**Medieval Samples (8th–16th Centuries CE)**			
**Archeological Sites**	**Chronology (Centuries)**	**Abbreviations and Grouping**	**References**
Colonna	8th–10th centuries CE	CO	[39]
Santa Severa	7th–15th centuries CE	SS	[43]
Allumiere	15th–16th centuries CE	AL	[40]
Cencelle	12th–15th centuries CE	CC	[41]
Piazza Madonna di Loreto	8th century CE	RMM	[44]
San Pancrazio	7th–8th centuries CE	RMM	[45]
Celio I	6th–9th centuries CE	RMM	[45]
Celio II	10th–11th centuries CE	RMM	[45]
Foro della Pace	10th–11th centuries CE	RMM	[45]
Gabii San Primitivo	10th–11th centuries CE	GSP	[45]

## Supplementary Materials

The following are available online, Figure S1: Empirical distribution of carbon and nitrogen isotopic values between sexes: (a) δ^13^C distribution in Roman Imperial populations; (b) δ^15^N distribution in Roman Imperial populations; (c) δ^13^C distribution in Medieval populations; (d) δ^15^N distribution in Medieval populations.

## Sample Availability

Samples of the compounds are available from the authors.

## References

[1] Makarewicz C.A., Sealy J. (2015). Dietary reconstruction, mobility, and the analysis of ancient skeletal tissues: Expanding the prospects of stable isotope research in archaeology. J. Archaeol. Sci..

[2] Schoeninger M.J., Larsen C.S. (2011). Diet reconstruction and ecology using stable isotope ratios. A Companion to Biological Anthropology.

[3] Sehrawat J.S., Kaur J. (2017). Role of stable isotope analyses in reconstructing past life-histories and the provenancing human skeletal remains: A review. Anthr. Rev..

[4] Curto A., Mahoney P., Maurer A.-F., Barrocas-Dias C., Fernandes T., Fahy G.E. (2019). Diet and disease in Tomar, Portugal: Comparing stable carbon and nitrogen isotope ratios between skeletons with and without signs of infectious disease. J. Archaeol. Sci..

[5] Redfern R.C., Dewitte S.N., Beaumont J., Millard A.R., Hamlin C. (2019). A new method for investigating the relationship between diet and mortality: Hazard analysis using dietary isotopes. Ann. Hum. Biol..

[6] Reitsema L.J., Holder S. (2018). Stable Isotope Analysis and the Study of Human Stress, Disease, and Nutrition. Bioarchaeol. Int..

[7] Scorrano G., Brilli M., Martinez-Labarga C., Giustini F., Pacciani E., Chilleri F., Scaldaferri F., Gasbarrini A., Gasbarrini G., Rickards O. (2014). Palaeodiet reconstruction in a woman with probable celiac disease: A stable isotope analysis of bone remains from the archaeological site of Cosa (Italy). Am. J. Phys. Anthr..

[8] Scorrano G. (2018). The Stable Isotope Method in Human Paleopathology and Nutritional Stress Analysis. Archaeol. Anthr. Open Access.

[9] Lightfoot E., Šlaus M., O’Connell T. (2012). Changing cultures, changing cuisines: Cultural transitions and dietary change in iron age, roman, and early medieval Croatia. Am. J. Phys. Anthr..

[10] Müldner G., Gilchrist R., Reynolds A. (2009). Investigating Medieval Diet and Society by Stable Isotope Analysis of Human Bone. Reflections: 50 Years of Medieval Archaeology, 1957–2007.

[11] Scorrano G., Baldoni M., Brilli M., Rolfo M.F., Fornaciari G., Rickards O., Martínez-Labarga C. (2019). Effect of Neolithic transition on an Italian community: Mora Cavorso (Jenne, Rome). Archaeol. Anthr. Sci..

[12] Zazpe I., Sanchez-Tainta A., Toledo E., Villegas A.S., Martinez-Gonzalez M.A. (2014). Dietary Patterns and Total Mortality in a Mediterranean Cohort: The SUN Project. J. Acad. Nutr. Diet..

[13] Biesbroek S., Verschuren W.M.M., Boer J.M.A., Van De Kamp M.E., Van Der Schouw Y.T., Geelen A., Looman M., Temme E.H.M. (2017). Does a better adherence to dietary guidelines reduce mortality risk and environmental impact in the Dutch sub-cohort of the European Prospective Investigation into Cancer and Nutrition?. Br. J. Nutr..

[14] Martínez-González M., Villegas A.S., DE Irala J., Marti A., Martínez J. (2002). Mediterranean Diet and Stroke: Objectives and Design of the SUN Project. Nutr. Neurosci..

[15] Hedges R.E., Stevens R.E., Richards M. (2004). Bone as a stable isotope archive for local climatic information. Quat. Sci. Rev..

[16] Killgrove K., Tykot R. (2013). Food for Rome: A stable isotope investigation of diet in the Imperial period (1st–3rd centuries AD). J. Anthr. Archaeol..

[17] Killgrove K., Tykot R.H. (2018). Diet and collapse: A stable isotope study of Imperial-era Gabii (1st–3rd centuries AD). J. Archaeol. Sci. Rep..

[18] Tykot R.H., Martini M., Milazzo M., Piacentini M. (2004). Stable isotope and diet: You are what you eat. Proceedings of the International School of Physiscs “Enrico Fermi” Course CLIV.

[19] Bocherens H., Drucker D. (2003). Trophic level isotopic enrichment of carbon and nitrogen in bone collagen: Case studies from recent and ancient terrestrial ecosystems. Int. J. Osteoarchaeol..

[20] Bogaard A., Heaton T.H.E., Poulton P., Merbach I. (2007). The impact of manuring on nitrogen isotope ratios in cereals: Archaeological implications for reconstruction of diet and crop management practices. J. Archaeol. Sci..

[21] Doppler T., Gerling C., Heyd V., Knipper C., Kuhn T., Lehmann M.F., Pike A.W.G., Schibler J. (2017). Landscape opening and herding strategies: Carbon isotope analyses of herbivore bone collagen from the Neolithic and Bronze Age lakeshore site of Zurich-Mozartstrasse, Switzerland. Quat. Int..

[22] Fraser R.A., Bogaard A., Heaton T., Charles M., Jones G., Christensen B.T., Halstead P., Merbach I., Poulton P.R., Sparkes D. (2011). Manuring and stable nitrogen isotope ratios in cereals and pulses: Towards a new archaeobotanical approach to the inference of land use and dietary practices. J. Arcaheol. Sci..

[23] Gron K.J., Gröcke D.R., Larsson M., Sørensen L., Larsson L., Rowley-Conwy P., Church M.J. (2017). Nitrogen isotope evidence for manuring of early Neolithic Funnel Beaker Culture cereals from Stensborg, Sweden. J. Archaeol. Sci. Rep..

[24] Göhring A., Mauder M., Vohberger M., Nehlich O., von Carnap-Bornheim C., Hilberg V., Kröger P., Grupe G. (2018). Palaeobiodiversity research based on stable isotopes: Correction of the sea spray effect on bone carbonate δ^13^C and δ^18^O by Gaussian Mixture Model clustering. Palaeogeogr. Palaeoclimatol. Palaeoecol..

[25] Hedges R.E., Reynard L.M. (2007). Nitrogen isotopes and the trophic level of humans in archaeology. J. Archaeol. Sci..

[26] Larsson M., Bergman J., Lagerås P. (2019). Manuring practices in the first millennium AD in southern Sweden inferred from isotopic analysis of crop remains. PLoS ONE.

[27] Makarewicz C.A. (2014). Winter pasturing practices and variable fodder provisioning detected in nitrogen (δ^15^N) and carbon (δ^13^C) isotopes in sheep dentinal collagen. J. Archaeol. Sci..

[28] Nehlich O., Fuller B.T., Marquez-Grant N., Richards M.P. (2012). Investigation of diachronic dietary patterns on the islands of Ibiza and Formentera, Spain: Evidence from sulphur stable isotope ratio analysis. Am. J. Phys. Anthr..

[29] Schulting R.J., Vaiglova P., Crozier R., Reimer P.J. (2017). Further isotopic evidence for seaweed-eating sheep from Neolithic Orkney. J. Archaeol. Sci. Rep..

[30] Szpak P. (2014). Complexities of nitrogen isotope biogeochemistry in plant-soil systems: Implications for the study of ancient agricultural and animal management practices. Front. Plant Sci..

[31] Reitsema L.J. (2013). Beyond diet reconstruction: Stable isotope applications to human physiology, health, and nutrition. Am. J. Hum. Biol..

[32] Beaumont J., Montgomery J. (2016). The Great Irish Famine: Identifying Starvation in the Tissues of Victims Using Stable Isotope Analysis of Bone and Incremental Dentine Collagen. PLoS ONE.

[33] Crowder K., Montgomery J., Gröcke D., Kori F. (2019). Childhood “stress” and stable isotope life-historied in Transylvania. Int. J. Osteoarchaeol..

[34] Fuller B.T., Fuller J.L., Sage N.E., Harris D.A., O’Connell T.C., Hedges R.E.M. (2005). Nitrogen balance and δ^15^N: Why you’re not what you eat during nutritional stress. Rapid Commun. Mass Spectrom..

[35] Garland C.J., Reitsema L.J., Larsen C.S., Thomas D.H. (2018). Early Life Stress at Mission Santa Catalina de Guale: An Integrative Analysis of Enamel Defects and Dentin Incremental Isotope Variation in Malnutrition. Bioarchaeol. Int..

[36] Katzenberg M.A., Lovell N.C. (1999). Stable isotope variation in pathological bone. Int. J. Osteoarchaeol..

[37] Mekota A.M., Grupe G., Ufer S., Cuntz U. (2006). Serian analysis of stable nitrogen and carbon isotopes in hair: Monitoring star-vation and recovery phases of patiens suffering from anorexia nervosa. Rapid Commun. Mass Spectrom..

[38] Toyne M.J., Turner B.L. (2020). Linking isotope analysis and paleopathology: An Andean perspective. Int. J. Paleopathol..

[39] Baldoni M., Nardi A., Muldner G., Lelli R., Gnes M., Ferraresi F., Meloni V., Cerino P., Greco S., Manenti G. (2016). Archaeo-biological reconstruction of the Italian medieval population of Colonna (8th–10th centuries CE). J. Archaeol. Sci. Rep..

[40] Baldoni M., Scorrano G., Gismondi A., D’Agostino A., Alexander M., Gaspari L., Vallelonga F., Canini A., Rickards O., Martínez-Labarga C. (2018). Who were the miners of Allumiere? A multidisciplinary approach to reconstruct the osteobiography of an Italian worker community. PLoS ONE.

[41] Baldoni M., Scorrano G., Alexander M., Stasolla F., Marsella L., Rickards O., Martínez-Labarga C. (2019). The medieval population of Leopoli-Cencelle (Viterbo, Latium): Dietary reconstruction through stable isotope analysis from bone proteins. J. Archaeol. Sci. Rep..

[42] De Angelis F., Varano S., Battistini A., Di Giannantonio S., Ricci P., Lubritto C., Facchin G., Brancazi L., Santangeli-Valenzani R., Catalano P. (2020). Food at the heart of the Empire: Dietary reconstruction for Imperial Rome inhabitants. Archaeol. Anthr. Sci..

[43] Gismondi A., Baldoni M., Gnes M., Scorrano G., D’Agostino A., Di Marco G., Calabria G., Petrucci M., Müldner G., Von Tersch M. (2020). A multidisciplinary approach for investigating dietary and medicinal habits of the Medieval population of Santa Severa (7th–15th centuries, Rome, Italy). PLoS ONE.

[44] Pescucci L., Battistini A., De Angelis F., Catalano P. (2013). Vivere al centro di Roma nell’VIII secolo D.C. Indicazioni antropologiche. Boll. Archeol. On-Line.

[45] Varano S., De Angelis F., Battistini A., Brancazi L., Pantano W., Ricci P., Romboni M., Catalano P., Gazzaniga V., Lubritto C. (2020). The edge of the Empire: Diet characterization of medieval Rome through stable isotope analysis. Archaeol. Anthr. Sci..

[46] Purcell N. (2003). The Way We Used to Eat: Diet, Community, and History at Rome. Am. J. Philol..

[47] Wilkins J.M., Hill S. (2006). Food in the Ancient World.

[48] Yardley J.C., Slater W.J. (1991). The symposium in Roman elegy. Dining in a Classical Context.

[49] Delgado A.M., Vaz Almeida M.D., Parisi S. (2017). Chemistry of the Mediterranean Diet.

[50] Garnsey P. (1999). Food and Society in Classical Antiquity.

[51] De Ligt L., Rosenstein N., Morstein-Marx R. (2006). The economy: Agrarian change during the second century. A companion to the Roman Republic.

[52] Spurr M.S. (1983). The cultivation of millet in Roman Italy. Pap. Br. Sch. Rome.

[53] Prowse T.L. (2001). Isotopic and Dental Evidence for Diet from the Necropolis of Isola Sacra (1st–3rd centuries AD), Italy. Ph.D. Dissertation.

[54] Kron G., Erdkamp P., Holleran C. (2002). Comparative perspectives on nutrition and social inequality in the Roman World. Diet and Nutrition in the Roman World.

[55] MacKinnon M.R. (2004). Production and Consumption of Animals in Roman Italy: Integrating the Zooarchaeological and teXtual Evidence.

[56] Brothwell D., Brothwell P. (1998). Food in Antiquity: A Survey of the Diet of Early Peoples.

[57] Marzano A. (2018). Fish and fishing in the Roman World. J. Marit. Archaeol..

[58] Rowan E. (2017). Bioarchaeological preservation and non-elite diet in the Bay of Naples: An analysis of the food remains from the Cardo V sewer at Roman site of Herculaneum. Environ. Arcaheol..

[59] Gismondi A., D’Agostino A., Di Marco G., Martínez-Labarga C., Leonini V., Rickards O., Canini A. (2020). Back to the roots: Dental calculus analysis of the first documented case of coeliac disease. Archaeol. Anthr. Sci..

[60] Gismondi A., D’Agostino A., Di Marco G., Scuderi F., De Angelis F., Rickards O., Catalano P., Canini A. (2020). Archaeobotanical record from dental calculus of a Roman individual affected by bilateral temporo-mandibular joint ankylosis. Quat. Int..

[61] King A. (1999). Diet in the Roman world: A regional inter-site comparison of the mammal bones. J. Rom. Archaeol..

[62] Cool H.E.M. (2006). Eating and Drinking in Roman Britain.

[63] Prowse T.L., Schwarcz H.P., Saunders S.R., Macchiarelli R., Bondioli L. (2004). Isotopic paleodiet studies of skeletons from the Imperial Roman-age cemetery of Isola Sacra, Rome, Italy. J. Archaeol. Sci..

[64] Prowse T.L., Schwarcz H.P., Saunders S.R., Macchiarelli R., Bondioli L. (2005). Isotopic evidence for age-related variation in diet from Isola Sacra, Italy. Am. J. Phys. Anthr..

[65] Prowse T.L., Saunders S.R., Schwarcz H.P., Garnsey P., Macchiarelli R., Bondioli L. (2008). Isotopic and dental evidence for infant and young child feeding practices in an imperial Roman skeletal sample. Am. J. Phys. Anthr..

[66] O’Connell T.C., Ballantyne R., Hamilton-Dyer S., Margaritis E., Oxford S., Pantano W., Millett M., Keay S.J. (2019). Living and dying at the Portus Romae. Antiquity.

[67] Rutgers L.V., van Strydonc M., Boudin M. (2009). Stable isotope data from the early Christian catacombs of ancient Rome: New in-sights into the dietary habits of Rome’s early Christians. J. Archaeol. Sci..

[68] Salesse K. (2015). Archéo-Biogéchimie Isotopique, Reconstitutions des Régimes Alimentaires et des Chémas de Mobilité, et Interactions Bioculturelles. Les Sépultures Plurielles de la Catacombe des Saints Pierre-Et Marcellin (Rome, Ier-Iie S. Ap. J.-C.): Les Sépultures Plurielles de la Région X de la Catacombe des Saints Pierre-Et-Marcellin (Rome, Ier-Iiie S. Ap. J.-C.). Archéologie et Préhistoire.

[69] Salesse K., Dufour E., Lebon M., Wurster C., Castex D., Bruzek J., Zazzo A. (2014). Variability of bone preservation in a confined environment: The case of the catacomb of Sts Peter and Marcellinius (Rome, Italy). Palaeogeogr. Palaeoclimatol. Palaeoecol..

[70] Killgrove K., Montgomery J. (2016). All roads lead to Rome: Exploring human migration to the eternal city through biochemistry of skeletons from two Imperial-Era cemeteries (1st–3rd c AD). PLoS ONE.

[71] De Angelis F., Veltre V., Varano S., Romboni M., Renzi S., Zingale S., Ricci P., Caldarini C., Di Giannantonio S., Lubritto C. (2020). Dietary and Weaning Habits of the Roman Community of Quarto Cappello del Prete (Rome, 1st–3rd Century CE). Environ. Archaeol..

[72] Montanari M., Montanari M. (2012). Medioevo vicino, Medioevo lontano. Gusti del Medioevo. I Prodotti, la Cucina, la Tavola.

[73] Alexander M.M., Gerrard C.M., Gutiérrez A., Millard A.M. (2015). Diet, Society, and Economy in Late Medieval Spain: Stable Isotope Evidence from Muslims and Christians from Gandía, Valencia. Am. J. Phys. Anthropol..

[74] Alexander M.M., Gutiérrez A., Millard A.R., Richards M.P., Gerrard C.M. (2019). Economic and socio-cultural consequences of changing political rule on human and faunal diets in medieval Valencia (c. fifth–fifteenth century AD) as evidenced by stable isotopes. Archaeol. Anthr. Sci..

[75] Ciaffi R., Lelli R., Müldner G., Stantcheva K., Fischetti A., Ghini G., Craig O., Milano F., Rickards O., Arcudi G. (2015). Palaeobiology of the medieval population of Albano (Rome, Italy): A combined morphological and bio-molecular approach. Int. J. Osteoarchaeol..

[76] Paladin A., Moghaddam N., Stawinoga A.E., Siebke I., DePellegrin V., Tecchiati U., Lösch S., Zink A. (2020). Early medieval Italian Alps: Reconstructing diet and mobility in the valleys. Archaeol. Anthr. Sci..

[77] Rolandsen G.L., Arthur P., Alexander M. (2019). A tale of two villages: Isotopic insight into diet, economy, cultural diversity and agrarian communities in medieval (11th–15th century CE) Apulia, Southern Italy. J. Archaeol. Sci. Rep..

[78] Duby G. (1975). Le Origini dell’Economia Europea. Guerrieri e Contadini nel Medioevo.

[79] Montanari M., Montanari M. (1988). Barbari e Romani. Alimentazione e Cultura nel Medioevo.

[80] Belcastro G., Rastelli E., Mariotti V., Consiglio C., Facchini F., Bonfiglioli B. (2007). Continuity or Discontinuity of the Life-Style in Central Italy during the Roman Imperial Age-Early Middle Ages Transition: Diet, Health, and Behavior. Am. J. Phys. Anthropol..

[81] Montanari M., Montanari M. (2012). Desiderio di carne. Gusti del Medioevo. I Prodotti, la Cucina, la Tavola.

[82] Alaica A.K., Schalburg-Clayton J., Dalton A., Kranioti E., Graziani Echávarri G., Pickard C. (2019). Variability along the frontier: Stable carbon and nitrogen isotope ratio analysis of human remains from the Late Roman-Early Byzantine cemetery site of Joan Planells, Ibiza, Spain. Archaeol. Anthropol. Sci..

[83] López-Costas O., Müldner G. (2016). Fringes of the empire: Diet and cultural change at the Roman to post-Roman transition in NW Iberia. Am. J. Phys. Anthropol..

[84] Tafuri M.A., Goude G., Manzi G. (2018). Isotopic evidence of diet variation at the transition between classical and post-classical times in Central Italy. J. Archaeol. Sci. Rep..

[85] Gismondi A., D’Agostino A., Canuti L., Di Marco G., Martínez-Labarga C., Angle M., Rickards O., Canini A. (2018). Dental calculus reveals dietary habits and medicinal plant use in the Early Medieval Italian population of Colonna. J. Archaeol. Sci. Rep..

[86] Iacumin P., Galli E., Cavalli F., Cecere L. (2014). C_4_-Consumers in Southern Europe: The Case of Friuli V.G. (NE-Italy) During Early and Central Middle Ages. Am. J. Phys. Anthropol..

[87] Reitsema L.J., Vercellotti G. (2012). Stable Isotope Evidence for Sex- and Status- Based Variations in Diet and Life History at Medieval Trino Vercellese, Italy. Am. J. Phys. Anthropol..

[88] Montanari M., Montanari M. (1993). A ciascuno il suo. La fame e l’abbondanza. Storia dell’Alimentazione in Europa.

[89] Montanari M. (1984). Campagne Medievali. Strutture Produttive, Rapporti di Lavoro, Sistemi Alimentari.

[90] Montanari M., Montanari M. (1988). Modelli di civiltà: Il consumo di cereali. Alimentazione e Cultura nel Medioevo.

[91] Montanari M., Montanari M. (1993). La svolta. La Fame e l’Abbondanza. Storia dell’Alimentazione in Europa.

[92] Montanari M., Montanari M. (2012). Profumo di civiltà: Il pane. Gusti del Medioevo. I Prodotti, la Cucina, la Tavola.

[93] Montanari M., Montanari M. (1993). L’Europa e il mondo. La Fame e l’Abbondanza. Storia dell’Alimentazione in Europa.

[94] Montanari M., Montanari M. (1988). Diete monastiche. Alimentazione e Cultura nel Medioevo.

[95] Barrett J.H., Richards M.P. (2004). Identity, gender, religion and economy: New isotope and radiocarbon evidence for marine resource intensification in early historic Orkney, Scotland, UK. Eur. J. Archaeol..

[96] Lanconelli A. (1985). Gli statuta pescivendulorum Urbis (1405). Note sul commerco di pesca a Roma fra XIV e XV secolo. Arch. Soc. Romana Stor. Patria.

[97] Lanconelli A. (1990). I Lavori alla Peschiera del Marta. Contributo alla Storia della Pesca nel Lazio Bassomedievale, Scritti in Memoria di Giuseppe Marchetti Longhi.

[98] Müldner G., Richards M.P. (2005). Fast or feast: Reconstructing diet in later medieval England by stable isotope analysis. J. Archaeol. Sci..

[99] Müldner G., Richards M.P. (2007). Stable isotopic evidence for 1500 years of human diet at the city of York, UK. Am. J. Phys. Anthropol..

[100] Nada Patrone A.M.N. (1981). Il Cibo del Ricco ed il Cibo del Povero: Contrinuto alla Storia Qualitativa dell’Alimentazione.

[101] Reitsema L.J., Crews D.E., Polcyn M. (2010). Preliminary ecidence for medieval Polish diet from carbon and nitrogen stable isotopes. J. Archaeol. Sci..

[102] Salamon M., Coppa A., McCormick M., Rubini M., Vargui R., Tuross N. (2008). The consilience of historical and isotopic approaches in recontructing the medieval Mediterranean diet. J. Archaeol. Sci..

[103] Sundman E.A. (2018). Masculinities and diet: An analysis of skeletal material from the Dominican priory in Medieval Västerås, Sweden. Nor. Arcahaeol. Rev..

[104] Woolgar C., Starkey D.J., Reid C., Ashcroft N. (2000). Take this penance now, and afterwards the fare will improve: Seafood and late medieval diet. England’s Sea Fisheries: The Commercial Sea Fisheries of England and Wales Since 1300.

[105] Zug Tucci H. (1985). Il Mondo Medievale dei Esci tra Realtà e Immaginazione. L’Uomo di Fronte al Mondo Animale nell’Alto Medioevo.

[106] Southern P. (2015). The third century: The nature of the problem. The Roman Empire from Severus to Constantine.

[107] Waters-Rist A.L., Katzenberg M.A. (2010). The effect of growth on stable nitrogen isotope ratios in subadult bone collagen. Int. J. Osteoarchaeol..

[108] Barrea L., Muscogiuri G., Frias-Toral E., Laudisio D., Pugliese G., Castellucci B., Garcia-Velasquez E., Savastano S., Colao A. (2020). Nutrition and immune system: From the Mediterranean diet to dietary supplementary through the microbiota. Crit. Rev. Food Sci. Nutr..

[109] Iddir M., Brito A., Dingeo G., Del Campo S.S.F., Samouda H., La Frano M.R., Bohn T. (2020). Strengthening the Immune System and Reducing Inflammation and Oxidative Stress through Diet and Nutrition: Considerations during the COVID-19 Crisis. Nutrients.

[110] Kau A.L., Ahern P.P., Griffin N.W., Goodman A.L., Gordon J.I. (2011). Human nutrition, the gut microbiome and the immune system. Nat. Cell Biol..

[111] Keusch G.T. (2003). The History of Nutrition: Malnutrition, Infection and Immunity. J. Nutr..

[112] Gombart A.F., Pierre A., Maggini S. (2020). A Review of Micronutrients and the Immune System–Working in Harmony to Reduce the Risk of Infection. Nutrients.

[113] Antonio M.L., Gao Z., Moots H.M., Lucci M., Candilio F., Sawyer S., Oberreiter V., Calderon D., Devitofranceschi K., Aikens R.C. (2019). Ancient Rome: A genetic crossroads of Europe and the Mediterranean. Science.

[114] Jongman W.M., Jacobs J.P., Goldewijk G.M.K. (2019). Health and wealth in the Roman Empire. Econ. Hum. Biol..

[115] De Angelis F., Veltre V., Romboni M., Di Corcia T., Scano G., Martínez-Labarga C., Catalano P., Rickards O. (2021). Ancient genomes from a rural site in Imperial Rome (1st–3rd cent. CE): A genetic junction in the Roman Empire. Ann. Hum. Biol..

[116] Caldarini C., Zavaroni F., Benassi V. (2015). Indicatori scheletrici di lavoro: Marcatori muscolo-scheletrici, artropatie e traumi. J. Hist. Med..

[117] Mosticone R., Pescucci L., Porreca F. (2015). Le condizioni di vita: Indicatori di stress aspecifici e affezioni dento-alveolari. J. Hist. Med..

[118] Wickham C. (2005). Framing the Early Middle Ages: Europe and the Mediterranean, 400–800.

[119] Barbehenn R., Chen Z., Karowe D.N., Spickard A. (2004). C_3_ grasses have higher nutritional quality than C_4_ grasses under ambient and elevated atmospheric CO_2_. Glob. Chang. Biol..

[120] Fernando N., Panozzo J., Tausz M., Norton R.M., Naumann N., Fitzgerald G.J., Seneweera S. (2014). Elevated CO_2_ alters grain quality of two bread wheat cultivars grown under different environmental conditions. Agric. Ecosyst. Environ..

[121] Fernando N., Panozzo J., Tausz M., Norton R.M., Fitzgerals G.J., Myers S., Walker C., Strangoulis J., Seneweera S. (2012). Wheat grain quality under increasing atmospheric CO_2_ concentrations in a semi-arid cropping system. J. Cereal Sci..

[122] Fernando N., Panozzo J., Tausz M., Norton R., Fitzgerald G., Seneweera S. (2012). Rising atmospheric CO_2_ concentration affects mineral nutrient and protein concentration of wheat grain. Food Chem..

[123] Jobe T.O., Rahimzadeh Karvansara P., Zenzen I., Kopriva S. (2020). Ensuring nutritious food under elevated CO_2_ Conditions: A care for improved C_4_ crops. Front. Plant Sci..

[124] Myers S.S., Zanobetti A., Kloog I., Huybers P., Leakey A.D.B., Bloom A.J., Carlisle E., Dietterich L.H., Fitzgerald G., Hasegawa T. (2014). Increasing CO_2_ threatens human nutrition. Nature.

[125] Soares J.C., Santos C.S., Carvalho S.M., Pintado M.M., Vasconcelos M.W. (2019). Preserving the nutritional quality of crop plants under a changing climate: Importance and strategies. Plant Soil.

[126] Ujiie K., Ishimaru K., Hirotsu N., Nagasaka S., Miyakoshi Y., Ota M., Tokida T., Sakai H., Usui Y., Ono K. (2019). How elevated CO_2_ affects our nutrition in rice, and how we can deal with it. PLoS ONE.

[127] Butz D.E., Casperson S.L., Whigham L.D. (2014). The emerging role of carbon isotope ratio determination in health research and medical diagnostics. J. Anal. At. Spectrom..

[128] DeWitte S.N., Agarwal S.C., Wesp J.K. (2017). Sex and frailty. Exploring Sex and Gender in Bioarchaeology.

[129] Roberts C.A. (2009). Human Remains in Archaeology: A Handbook.

[130] Wells C. (1975). Ancient obstetric hazards and female mortality. Bull. N. Y. Acad. Med..

[131] Chen H., Wang P., Han Y., Ma J., Troy F.A., Wang B. (2012). Evaluation of dietary intake of lactating women in China and its potential impact on the health of mothers and infants. BMC Women Health.

[132] Engidaw M.T., Gebremariam A.D., Tiruneh S.A., Asnakew D.T., Abate B.A. (2019). Chronic Energy Deficiency and its Associated Factors Among Lactating Women in Debre Tabor General Hospital, Northcentral Ethiopia. J. Fam. Med. Health Care.

[133] Hollis B.W., Wagner C.L. (2004). Vitamin D requirements during lactation: High-dose maternal supplementation as therapy to prevent hypovitaminosis D for both the mother and the nursing infant. Am. J. Clin. Nutr..

[134] Lovejoy C.O., Meindl R.S., Pryzbeck T.R., Mensforth R.P. (1985). Chronological metamorphosis of the auricular surface of the ilium: A new method for the determination of adult skeletal age at death. Am. J. Phys. Anthr..

[135] Brooks S., Suchey J.M. (1990). Skeletal age determination based on the os pubis: A comparison of the Acsádi-Nemeskéri and Suchey-Brooks methods. Hum. Evol..

[136] Işcan M.Y., Loth S.R., Wright R.K. (1984). Metamorphosis at the sternal rib end: A new method to estimate age at death in white males. Am. J. Phys. Anthr..

[137] Işcan M.Y., Loth S.R., Wright R.K. (1985). Age estimation from the rib by phase analysis: White females. J. Forensic Sci..

[138] Meindl R.S., Lovejoy C.O. (1985). Ectocranial suture closure: A revised method for the determination of skeletal age at death based on the lateral-anterior sutures. Am. J. Phys. Anthr..

[139] Brothwell D.R. (1981). Digging up Bones: The Excavation, Treatment, and Study of Human Skeletal Remains.

[140] Lovejoy C.O. (1985). Dental wear in the Libben population: Its functional pattern and role in the determination of adult skeletal age at death. Am. J. Phys. Anthropol..

[141] Ubelaker D.H. (1989). Human Skeletal Remains: Excavation, Analysis, Interpretation.

[142] Fazekas I.G., Kósa F. (1978). Forensic Fetal Osteology.

[143] Stloukal M., Hanáková H. (1978). Die Länge der Längsknochen altslawischer Bevölkerungen-Unter besonderer Berücksichtigung von Wachstumsfragen. Homo.

[144] Scheuer L., Black S. (2000). Developmental Juvenile Osteology.

[145] Scheuer L., Black S. (2004). Juvenile Osteology.

[146] Acsádi G., Nemeskéri J. (1970). History of Human Life Span and Mortality.

[147] Ferembach D., Schwidetzky I., Stloukal M. (1979). Recommandations pour determiner l’âge et le sexe sur le squelette. Bull. Mém. Soc. Anthropol. Paris.

[148] Phenice T.W. (1969). A newly developed visual method of sexing the os pubis. Am. J. Phys. Anthropol..

[149] Cox D.R. (1972). Regression Models and Life-Tables. J. R. Stat. Soc. Series B Stat. Methodol..

[150] Cox D.R. (1975). Partial Likelihood. Biometrika.

[151] Therneau T.M., Grambsch P.M., Fleming T.R. (1990). Martingale-Based Residuals for Survival Models. Biometrika.

[152] Grambsch P.M., Therneau T.M. (1994). Proportional hazards tests and diagnostics based on weighted residuals. Biometrika.

[153] Schoenfeld D. (1982). Partial residuals for the proportional hazards regression model. Biometrika.

[154] Hall P., Opsomer D. (2005). Theory for Penalised Spline Regression. Biometrika.

