# Supplementary material for: How Does Diet Influence Our Lives? Evaluating the Relationship between Isotopic Signatures and Mortality Patterns in Italian Roman Imperial and Medieval Periods
Source: Molecules. 2021 Jun 25;26(13):3895. doi: 10.3390/molecules26133895 (PMC8271375; doi:10.3390/molecules26133895)
Supplement: Supplementary file 1 [file molecules-26-03895-s001.zip › molecules-1245955-supplementary.pdf]

Supplementary Material

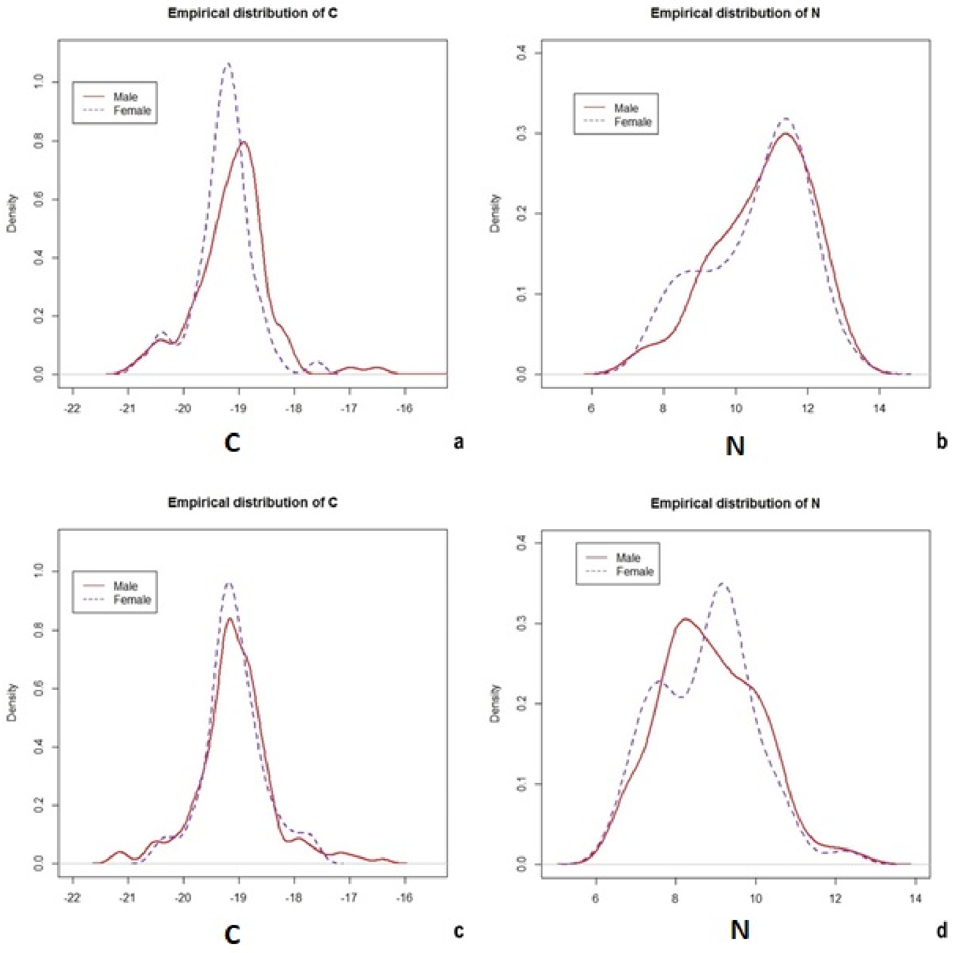

Figure S1. Empirical distribution of carbon and nitrogen isotopic values between sexes: (**a**) $\delta^{13}C$ distribution in Roman Imperial populations; (**b**) $\delta^{15}N$ distribution in Roman Imperial populations; (**c**) $\delta^{13}C$ distribution in Medieval populations; (**d**) $\delta^{15}N$ distribution in Medieval populations.
